# p75NTR antibody-conjugated microspheres: an approach to guided tissue regeneration by selective recruitment of endogenous periodontal ligament cells

**DOI:** 10.3389/fbioe.2024.1338029

**Published:** 2024-01-31

**Authors:** Xuqiang Zou, Bo Xie, Xuelian Peng, Mingjie Lu, Dan Xu, Hongyan Yuan, Yixin Zhang, Di Wang, Manzhu Zhao, Rui Liu, Xiujie Wen

**Affiliations:** ^1^ Department of Orthodontics, School of Stomatology, Southwest Medical University, Luzhou, China; ^2^ Chongqing Key Laboratory for Oral Diseases and Biomedical Sciences, Chongqing Municipal Key Laboratory of Oral Biomedical Engineering of Higher Education, College of Stomatology, Chongqing Medical University, Chongqing, China; ^3^ Department of Stomatology, Daping Hospital, Third Military Medical University (Army Medical University), Chongqing, China

**Keywords:** p75NTR, antibody conjugation, periodontal ligament cells, chitosan microspheres, nano-hydroxyapatite, cell recruitment

## Abstract

Repairing defects in alveolar bone is essential for regenerating periodontal tissue, but it is a formidable challenge. One promising therapeutic approach involves using a strategy that specifically recruits periodontal ligament cells (PDLCs) with high regenerative potential to achieve *in situ* regeneration of alveolar bone. In this study, we have created a new type of microsphere conjugated with an antibody to target p75 neurotrophin receptor (p75NTR), which is made of nano-hydroxyapatite (nHA) and chitosan (CS). The goal of this design is to attract p75NTR^+^hPDLCs selectively and promote osteogenesis. *In vitro* experiments demonstrated that the antibody-conjugated microspheres attracted significantly more PDLCs compared to non-conjugated microspheres. Incorporating nHA not only enhances cell adhesion and proliferation on the surface of the microsphere but also augments its osteoinductive properties. Microspheres effectively recruited p75NTR^+^ cells at bone defect sites in SD rats, as observed through immunofluorescent staining of p75NTR antibodies. This p75NTR antibody-conjugated nHA/CS microsphere presents a promising approach for selectively recruiting cells and repairing bone defects.

## 1 Introduction

The alveolar bone is a highly active component in the skeletal system that undergoes continuous metabolism and remodeling. Its integrity may be compromised by inflammation, trauma, tumors, and other factors (Barone et al., 2013; Petrovic et al., 2018; Rams et al., 2018). As such, restoring alveolar bone defects is a great concern in oral medicine with crucial implications for the treatment of oral diseases and the maintenance of oral health overall. Given its potential for repair and cost-efficiency, bone tissue engineering, which uses cells, scaffolds, and growth factors to create functional composite materials, seems like a promising approach for regeneration of alveolar bone tissues (Gjerde et al., 2018; Wang et al., 2018). However, issues related to exogenous cells, which include potential tumorigenicity, unreliable differentiation efficiency, and challenges in maintaining cell viability (Kucia and Ratajczak, 2006; Chen et al., 2011; Lin et al., 2019), have motivated scholars to explore *in situ* tissue engineering methods that employ endogenous cells (Hench et al., 2004; Anitua et al., 2010; Ko et al., 2013; Hu et al., 2017; Ullah et al., 2019). Recruiting cells with high regenerative activity in the damaged area and its surrounding tissues and promoting their differentiation into target tissue is crucial for achieving *in situ* bone regeneration. This approach avoids the drawbacks of exogenous cell implantation. Therefore, it is important to recruit a sufficient number of cells with high activity in bone regeneration at the bone defect site.

Human periodontal ligament cells (hPDLCs) are a heterogeneous cell population found within the periodontal ligament. They exhibit multidirectional differentiation potential and strong self-renewal ability (Zhang et al., 2014), making them the main seed cells for periodontal tissue engineering (Tsumanuma et al., 2011). However, due to the complexity of this heterogeneous group, their biological characteristics vary. Further isolation of hPDLCs with high regenerative potential can enhance their application in bone tissue engineering. The p75 neurotrophin receptor (p75NTR), a membrane protein, belongs to the tumor necrosis factor receptor superfamily and serves as a specific marker for stem cells derived from the cranial neural crest (CNC) (Park et al., 2010; Hauser et al., 2012; Akiyama et al., 2014). Research indicates that CNC-derived stem cells are partly retained in the periodontal ligament after lineage evolution. These cells continue to express p75NTR and possess a high differentiation potential (Mantesso and Sharpe, 2009; Tomokiyo et al., 2012). Past research has demonstrated that p75NTR^+^hPDLCs display stem cell features and have a significant potential for osteogenic differentiation (Li et al., 2020). Furthermore, p75NTR plays an important part in cell migration (Anton et al., 1994; Jiang et al., 2015). Therefore, it is postulated that the p75NTR antibody may function as an effective factor in selectively attracting hPDLCs with advanced regenerative potential for *in vivo* bone regeneration.

Chitosan (CS) is one of the most recognized scaffold materials due to its molecular structure similarity to glycosaminoglycan (GAG), which is a crucial component of the extracellular matrix (ECM). In addition, CS exhibits excellent biocompatibility, biodegradability, antimicrobial properties, and affordability (Kumar et al., 2004; Bano et al., 2017; Omer et al., 2021). CS microspheres present a sufficiently large surface area for cell adhesion and enable direct exchange with surrounding substances, making them an encouraging cell carrier (Yang et al., 2022). However, the restricted mechanical properties and osteoinductivity of CS limit its use in bone tissue engineering (Janković et al., 2013; Lavanya et al., 2020; Brun et al., 2021). Therefore, researchers often combine CS with bioactive materials. Nano-hydroxyapatite (nHA), which has a structure similar to normal bone tissue, not only improves osteoblast adhesion and proliferation but also demonstrates osteoinductivity and osteoconductivity, making it a frequently used bone graft substitute (Šupová, 2009; Dutta et al., 2015; Salgado et al., 2016; Kuang et al., 2019). Furthermore, nano-hydroxyapatite is gradually being incorporated into oral research, including studies on the proliferation and differentiation of dental pulp stem cells, periodontal tissue regeneration, and jawbone regeneration (Uehara et al., 2016; Xu et al., 2019; Yu et al., 2019).

The study describes the fabrication of nHA/CS microspheres through electrostatic spray method, followed by grafting biotin-modified p75NTR antibodies onto their surface via biotin-Streptavidin (SAV) pairing. This resulted in the formation of p75NTR antibody-conjugated nHA/CS microspheres. Furthermore, we evaluated the capacity of the novel nHA/CS microspheres to selectively recruit hPDLCs and facilitate osteogenesis in order to assess their potential for use in bone tissue engineering.

## 2 Materials and methods

### 2.1 Animals

All male nude mice and SD rats were obtained from the Southwest Medical University Laboratory Animal Center. All animals were kept under standard conditions in the Southwest Medical University Animal Laboratory. Six-week-old male nude mice were used to observe the effect of microspheres on inducing cell mineralization *in vivo*, and 8-week-old SD rats were used to prepare alveolar bone defects. All animal experiments were approved by the Ethics Committee of Animal Experiments of Southwest Medical University (authorization number: 201903-187).

### 2.2 Preparation of p75NTR antibody-conjugated nHA/CS microspheres

#### 2.2.1 Preparation of nano-hydroxyapatite (nHA)/chitosan (CS) microspheres

Acetic acid (Alfa Aesar, Heysham, UK) was mixed with deionized water to prepare a solution of 1% v/v acetic acid. Then, 1 g of chitosan powder (Aladdin, Shanghai, China) was dissolved in the acetic acid solution at a concentration of 1% w/v, followed by the addition of 1 g of nano-hydroxyapatite (Aladdin). The mixture was sonicated for 10 min to prepare a 1% w/v solution of nano-hydroxyapatite (nHA)/chitosan (CS). Subsequently, the nHA/CS solution was injected into a 1.0 M sodium hydroxide solution (Aladdin), using a jet apparatus comprising an electrostatic spray system (SS-X3, Yongkang Leye, Beijing, China) and a 5 mL syringe, resulting in the formation of nHA/CS microspheres. The collected microspheres were washed repeatedly with deionized water and then filtered through a 100-μm filter (Corning, New York, United States). After sterilization with 75% ethanol, the microspheres were washed with phosphate buffered saline (PBS) (Biosharp, Anhui, China) and stored in PBS solution at 4°C for future use.

#### 2.2.2 Modification of nHA/CS microsphere surface with p75NTR antibody

A mixture of PBS and DMSO solution (SIGMA-ALDRICH, St. Louis, United States) was prepared in a 3:1 ratio. (+)-biotin N-hydroxysuccinimide ester (biotin-NHS; SIGMA-ALDRICH) was added to the mixture to prepare a 1 mg/mL solution. The solution was stirred with the microspheres at room temperature for 4 h to allow biotin to adhere to the surface of the microspheres, and then washed three times with the PBS solution. Subsequently, the reacted microspheres were immersed in a solution of Streptavidin (SAV; Roche, Mannheim, Germany) at a concentration of 50 μg/mL at room temperature for 15 min, and the unreacted SAV was washed with PBS. The biotinylated p75NTR antibody (1 mg/mL) (Bioss, Beijing, China) was then incubated with the microspheres at room temperature for 30 min. After washing three times with PBS, microspheres with surface-modified p75NTR antibody were obtained. The results of antibody conjugation were evaluated using secondary-labeled antibody Cy3 (Invitrogen, Waltham, United States). Observations were conducted under a fluorescence microscope (IX73, Olympus, Japan) to examine the microspheres.

#### 2.2.3 Scanning electron microscopy

The CS microspheres and nHA/CS microspheres were imaged using a scanning electron microscope (SU8100, Hitachi, Japan). The samples were placed on a double-sided conductive carbon tape and inserted into the sample stage of an ion sputter coater (MC1000, HITACHI, Japan) for gold spraying. The imaging was performed at an acceleration voltage of 3 kV.

### 2.3 Cell isolation and culture

The tissues were taken after obtaining approval from the Ethics Committee of the Affiliated Stomatology Hospital of Southwest Medical University and informed consent from the patients. Primary hPDLCs were isolated from the premolars of patients aged 18–25 years who underwent orthodontic treatment. Periodontal ligaments were scraped from the root surface, digested with 2 mg/mL collagenase I (Solarbio, Beijing, China) at 37°C for 30 min, and neutralized with α-minimum essential medium (α-MEM; Gibco, Waltham, United States) supplemented with 10% fetal bovine serum (FBS; Gibco). The cell suspension was centrifuged at 1,000 rpm for 5 min, and the cell pellet was resuspended in α-MEM containing 10% FBS and 1% penicillin/streptomycin (Gibco). The cells were then cultured at 37°C in a humidified incubator with 5% CO_2_. The culture medium was routinely refreshed every 3 days. When 80% confluency was reached, the cells were dissociated and passed using trypsin (Gibco).

### 2.4 Flow cytometry analysis

Trypsin was used to digest the third-passage hPDLCs, which were subsequently resuspended in PBS. The Alexa Fluor 647-labeled mouse p75NTR antibody (BD Biosciences, New Jersey, United States) was incubated at room temperature for 30 min, with the Alexa Fluor 647-labeled mouse IgG1 antibody (BD Biosciences) serving as an isotype control. Subsequently, the specimens were analyzed by flow cytometry.

### 2.5 Immunofluorescence assay

Third-passage cells (2 × 10^5^) were inoculated in confocal culture dishes and incubated for 12 h. The cells were then fixed with 4% paraformaldehyde (Biosharp) for 30 min, followed by blocking with 2% bovine serum albumin (BSA) (Biosharp) at 4°C for 1 h. Subsequently, the cells were incubated overnight with rabbit anti-human p75NTR primary antibody (1:200; Abcam, Cambridge, United States) at 4°C and incubated with goat anti-rabbit IgG- Alexa Fluor 647 secondary antibody (1:500; Abcam) at 37 °C for 1 h. Finally, the specimens were counterstained with DAPI (Solarbio, Beijing, China) and observed under a fluorescence microscope.

### 2.6 Cell capture experiment

Cells were digested with trypsin and resuspended in medium at a concentration of 10^6^ cells/mL. The fluorescent dye, 1, 1′-Dioctadecyl-3, 3, 3′, 3′-tetramethylindocarbocyanine perchlorate (DiI; Beyotime, Shanghai, China), was added at a concentration of 5 μL/mL. After incubation for 20 min, centrifugation was performed three times to remove unreacted dye. Subsequently, the microspheres were counted under the optical microscope and adjusted to 200 microspheres per well in a 6-well low-adhesion plate (Corning) to co-incubate with the pre-stained cells at 37°C for 6 h. After incubation, cells not captured were filtered using a 70-μm cell strainer (Corning). A fluorescence microscope was used to observe the microspheres and to evaluate cell capture results. The CellTiter 96^®^ AQueous One Solution Cell Proliferation Assay (MTS; Promega, Madison, United States) was then used to quantitatively analyze the experiment results, following the manufacturer’s guidelines.

### 2.7 Flow cytometric analysis of captured cells

Cell-containing microspheres were digested by trypsin, followed by the addition of a complete culture medium. Microspheres were filtered using a 70-μm mesh filter, and subsequently, the cells were grown in T25 culture flasks (JetBiofil, Guangzhou, China). The culture medium was refreshed every 3 days. Once the cell confluence attained 80%, the expression of p75NTR was evaluated via flow cytometry, according to the procedures described above.

### 2.8 Cytoskeleton and nuclear staining experiment

The captured cells were further cultured in 6-well plates. At day 1, 10, and 21, microspheres with cells were fixed with 4% paraformaldehyde for 30 min, followed by a 2-h blockage at room temperature using 2% BSA. The specimens were then stained with FITC-phalloidin (5 μg/mL; Solarbio) for 20 min, and washed three times with the PBS solution to remove any unbound phalloidin. Afterward, DAPI staining was applied at a concentration of 10 μg/mL for 5 min before being observed under a fluorescence microscope.

### 2.9 MTS assay

After cell capture, 50 microspheres of each group were placed in 100 μL complete cell culture medium on 96-well plates. Blank wells containing only culture medium serve as controls. Each well was supplemented with 20 µL MTS solution (1.90 mg/mL; Promega, Madison, United States) and then cultured in a 37°C incubator with 5% CO_2_ for 3 h. Subsequently, 100 µL culture medium was taken from each well and transferred to a new 96-well plate. The absorbance was measured at 490 nm by a microplate reader (Bio Tek, Winooski, Vermont, United States). The results were obtained by subtracting the average absorbance of blank wells from the absorbance of each sample well.

### 2.10 Alkaline phosphatase (ALP) analysis

Each well of a 6-well plate was seeded with 200 microspheres and 5 × 10^4^ cells, followed by the addition of 2 mL of complete growth medium for co-cultivation. Once the cells reached 50% confluence, the osteogenic induction medium (comprising 5% FBS, 2% penicillin-streptomycin, 10 μmol/L dexamethasone, 50 μg/mL ascorbic acid, and 10 mmol/L β-glycerophosphate in α-MEM medium) was added. The osteogenic induction medium was replaced every 3 days. After 7 days of induction, the specimens were washed thrice with PBS, fixed for 30 min in 4% paraformaldehyde solution, and stained with an ALP staining kit (Beyotime) for 30 min. After washing twice with double distilled water, the specimens were observed by optical microscope. For quantitative analysis of ALP activity, detection was performed using an ALP assay kit (Beyotime). In brief, 1% Triton X-100 (Beyotime) was added to the 6-well plate for 20 min at room temperature. Then the cell lysate was extracted by centrifugation and transferred to a new 96-well plate. The relevant procedures were followed according to the instructions of the ALP assay kit, and the absorbance was measured at 405 nm using a microplate reader. All samples were normalized to total protein concentration by BCA Protein Assay Kit (Beyotime).

### 2.11 Quantitative reverse transcription (qRT)-PCR experiment

Cells were co-cultured with microspheres and osteogenically induced for 7 days, after which total RNA was extracted using Trizol reagent (Invitrogen, Waltham, United States). Subsequently, the RNA was reverse-transcribed into cDNA using the PrimeScript Reverse Transcription Kit (Takara, Tokyo, Japan). qPCR was performed using the SYBR Premix ExTaq Kit (Takara) to detect the expression levels of *ALP*, *RUNX2*, and *COL1.* The primer sequences are listed in Table 1, and the relative expression levels were calculated using the 2^-△△Ct^ method.

**TABLE 1:** Primer sequences used in this study.

mRNA name	Forward primers (5′–3′)	Reverse primers (5′–3′)	Product size (bp)
*Gapdh*	GTG​GAC​CTG​ACC​TGC​CGT​CTA​G	GTG​TCG​CTG​TTG​AAG​TCA​GAG​GAG	143
*p75NTR*	CAT​CCC​TGT​CTA​TTG​CTC​CAT​C	GAG​TTT​TTC​TCC​CTC​TGG​TGG	151
*ALP*	CTG​GTA​CTC​AGA​CAA​CGA​GAT​G	GTC​AAT​GTC​CCT​GAT​GTT​ATG​C	94
*RUNX2*	AGG​CAG​TTC​CCA​AGC​ATT​TCA​TCC	TGG​CAG​GTA​GGT​GTG​GTA​GTG​AG	150
*COL1*	GAG​GGC​CAA​GAC​GAA​GAC​ATC	CAG​ATC​ACG​TCA​TCG​CAC​AAC	140

### 2.12 Calcification deposition in nude mice experiment

Before subcutaneous implantation, cells and microspheres were cultured under osteogenic conditions for 2 weeks. Six-week-old male nude mice were anesthetized with 50 mg/kg of pentobarbital sodium injected intraperitoneally. After anesthesia, a longitudinal 1 cm skin incision was made on the back of each mouse, creating a subcutaneous pouch, into which cells and microspheres were implanted. Three weeks post-operation, the mice were euthanized by CO_2_ inhalation, and the implants were isolated by dissection. They were fixed in 4% paraformaldehyde solution at room temperature for 5 days, followed by embedding the implants in paraffin. Sections (5 µm) were prepared and stained with Alizarin Red S solution (Cyagen, Guangzhou, China) for 10 min. After washing twice with PBS, the specimens were observed by optical microscope to identify the formation of calcified matrix.

### 2.13 *In situ* recruitment of p75NTR^+^ cells

Eight-week-old SD rats were intraperitoneally anesthetized with pentobarbital sodium (50 mg/kg). An alveolar bone defect was created in the root bifurcation area of maxillary first molar palatal side using a circular carbide bur of 2.3 mm diameter (8# CARBIDE burs, Mani, Japan). Subsequently, the microspheres were meticulously implanted in the defective area of the maxillary alveolar bone, followed by a cautious suture of the mucous membrane to avoid any potential leakage of microspheres. On day 7, the rats were euthanized by CO_2_ inhalation. The alveolar bone tissues were collected and fixed in 4% paraformaldehyde solution. Microspheres were collected from the defect area. After blocking with 2% BSA for 1 h at room temperature, the samples were incubated overnight at 4°C with rabbit anti-rat p75NTR primary antibody (1:200; Absin, Shanghai, China) and incubated with goat anti-rabbit IgG-Alexa Fluor 488 secondary antibody (1:500; Abcam) at 37°C for 1 h, followed by incubation with DAPI for 5 min to stain the cell nuclei. Subsequently, the cells were observed under a fluorescence microscope (IX73, Olympus, Japan).

### 2.14 Statistical analysis

Data are expressed as mean ± standard deviation. Statistical comparisons were performed by one-way ANOVA with IBM SPSS Statistics for Windows version 17.0 software (IBM Corp., Armonk, N.Y., United States). One-way ANOVA with Tukey’s *post hoc* test was used for multiple comparisons. *p <* 0.05 was considered statistically significant.

## 3 Results

### 3.1 Preparation of p75NTR antibody-conjugated microspheres

**FIGURE 1:**
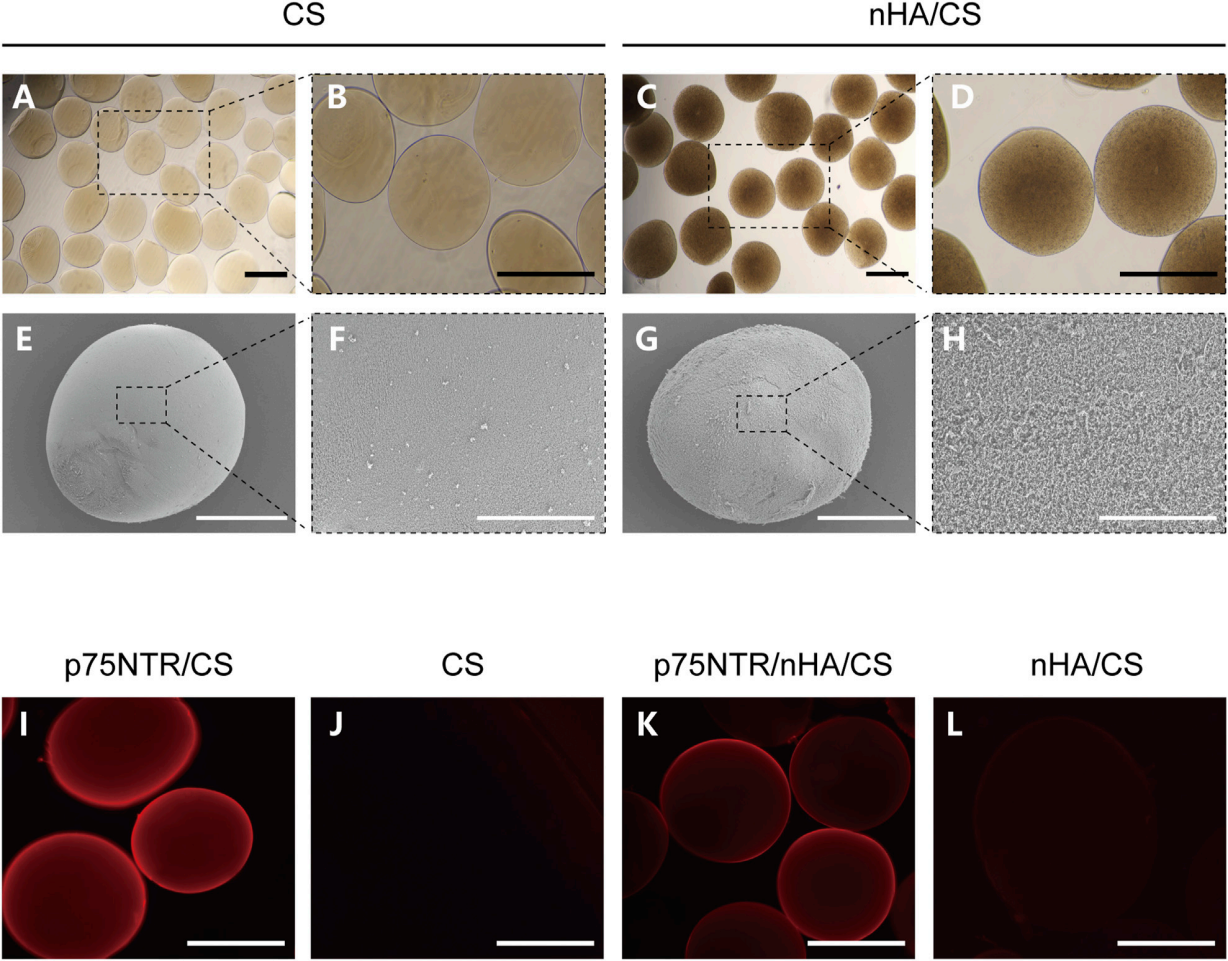
Preparation of microspheres conjugated with p75NTR antibody. **(A–D)** The optical microscopy image of the CS and nHA/CS microspheres. **(E–H)** SEM images of CS and nHA/CS microspheres. **(I–L)** Fluorescence images of p75NTR/CS, CS, p75NTR/nHA/CS and nHA/CS microspheres after incubation with a secondary-labeled antibody Cy3. Scale bars, 400 μm **(A–D)**, and **(I–L)**, 200 μm **(E,G)**, and 50 μm **(F,H)**.

Minute droplets of the CS and nHA/CS solution were generated by electrostatic spray method and subsequently solidified into microspheres through the sodium hydroxide solution. Microspheres that were too small for cells to attach and expand were filtered using a 100 μm filter. Under a light microscope, the CS microspheres appeared transparent, whereas those containing nHA turned brown (Figures 1A–D). Scanning electron microscopy revealed that the surface of nHA/CS microspheres (Figures 1G, H) was rougher than CS microspheres (Figures 1E, F).

The conjugation of the biotin-p75NTR antibody to the microspheres was verified by a secondary-labeled antibody Cy3. Both the p75NTR/CS and p75NTR/nHA/CS microspheres showed red fluorescence under the fluorescent microscope (Figures 1I, K), whereas no fluorescence was observed in the CS and nHA/CS groups (Figures 1J, L). These results implied that the microspheres were successfully and specifically conjugated with the p75NTR antibody. The diameters of microspheres ranged approximately from 100 to 540 μm, and the histogram of particle size distribution is shown in Supplementary Figure S1. The diameters of the CS group, nHA/CS group, p75NTR/CS group, and p75NTR/nHA/CS group were 376.60 ± 66.43 μm, 370.03 ± 68.90 μm, 369.43 ± 66.17 μm, and 370.48 ± 68.30 μm, respectively, indicating that there was no significant difference in the diameter size of the microspheres among these groups (*p* > 0.05).

### 3.2 Expression of p75NTR in hPDLCs

**FIGURE 2:**
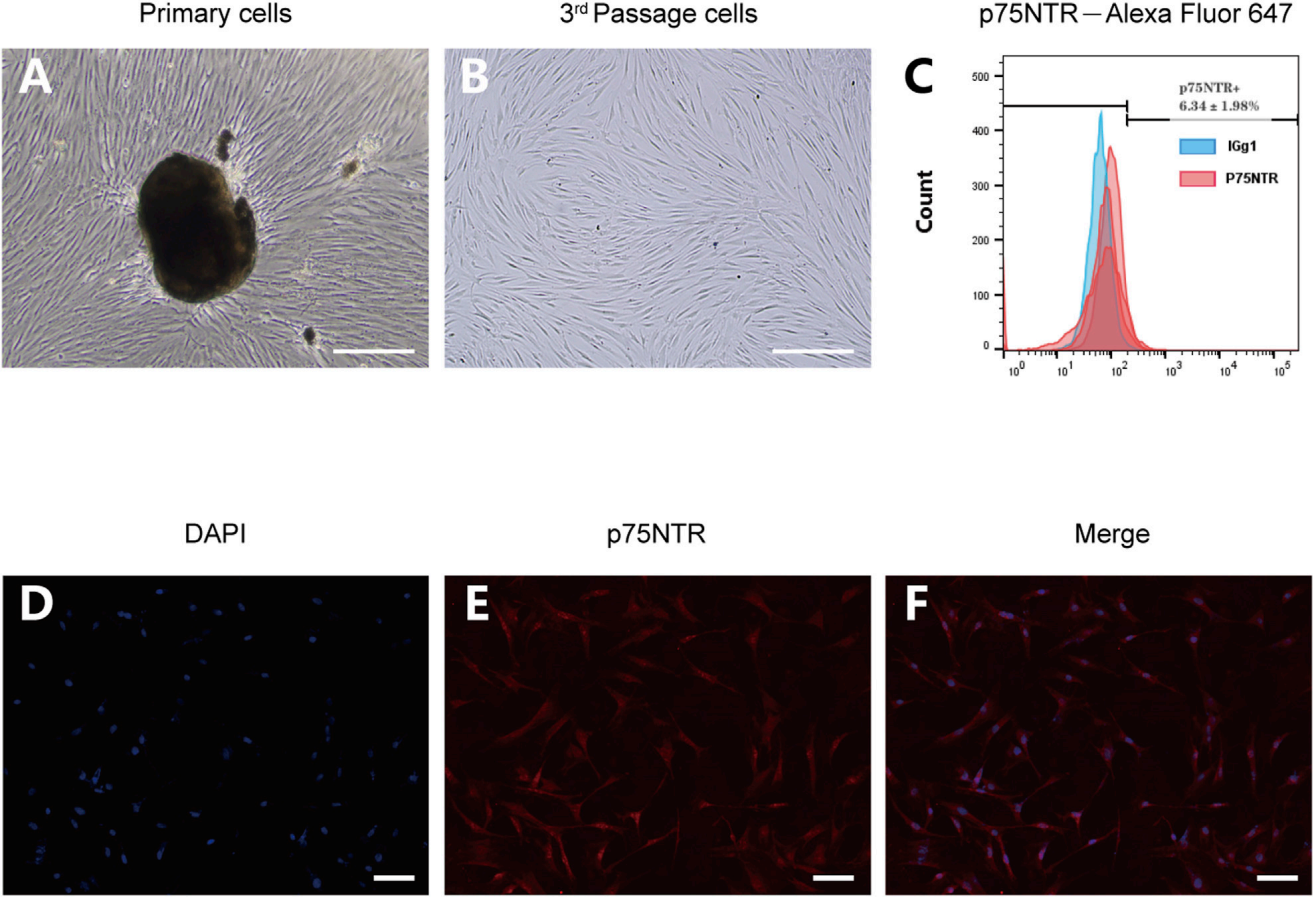
The expression of p75NTR in hPDLCs. **(A,B)** Primary and third-passage hPDLSCs were observed by optical microscopy Scale bar = 100 μm. **(C)** Flow cytometry analysis of hPDLSCs after incubation with Alexa Fluor 647-p75NTR antibody and Alexa Fluor 647-IgG1 isotype control antibody (n = 3). **(D–F)** Immunofluorescence assays of hPDLSCs stained with p75NTR (red) and DAPI (blue). Scale bar = 50 μm.

Isolated and cultured hPDLCs exhibited an elongated spindle morphology (Figures 2A, B). Both immunofluorescence and flow cytometry methods were employed to detect the expression of p75NTR in hPDLCs. Following staining with p75NTR fluorescent antibodies, red fluorescence was visible in the cells (Figures 2E, F). Flow cytometry results further demonstrated that the expression rate was 6.34% ± 1.98% (Figure 2C).

### 3.3 *In vitro* cell capture experiment

**FIGURE 3:**
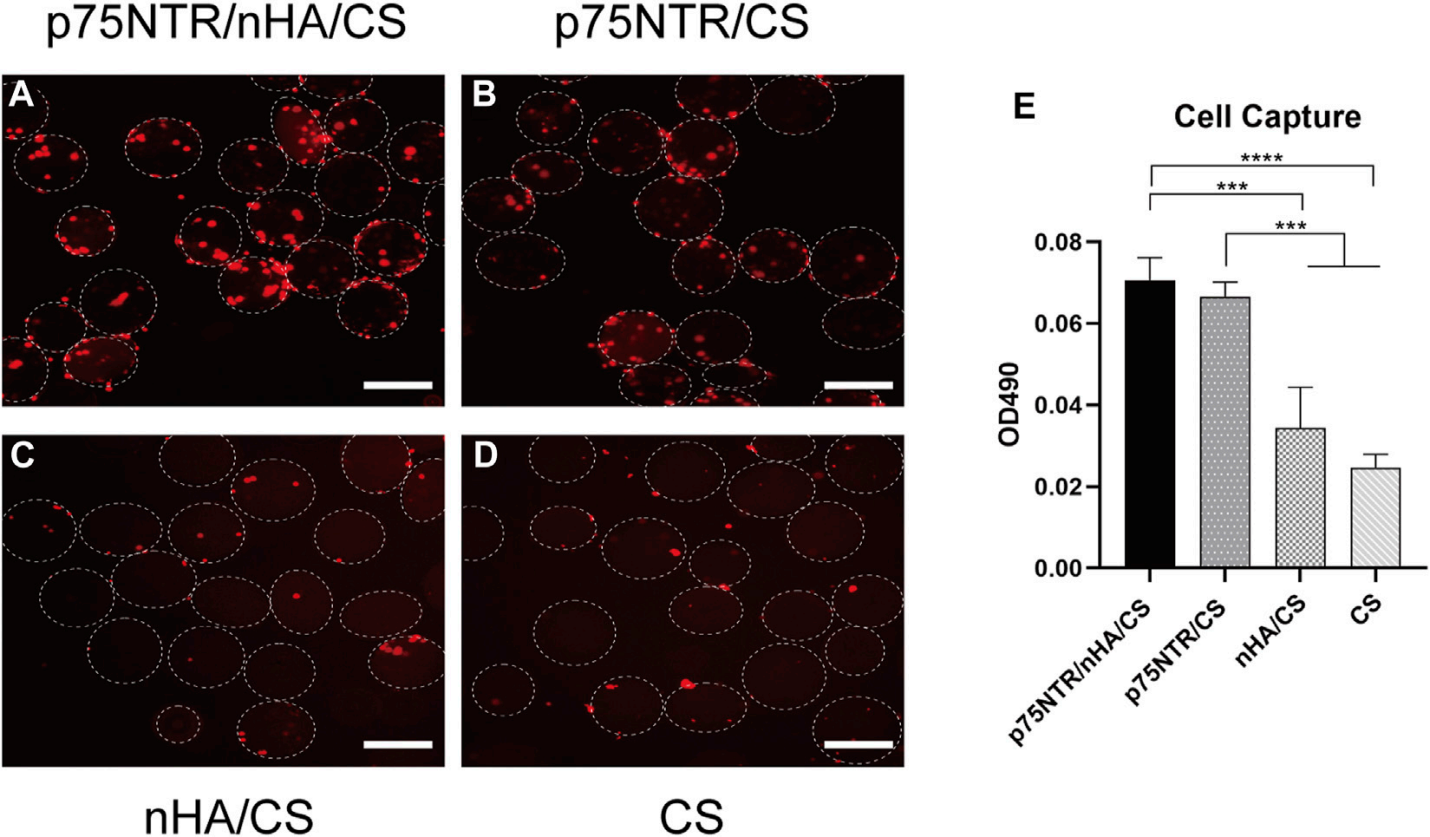
Ability of microspheres in capturing hPDLCs *in vitro*. Fluorescence image of captured hPDLCs (red) on p75NTR/nHA/CS microspheres **(A)**, p75NTR/CS microspheres **(B)**, nHA/CS microspheres **(C)**, and CS microspheres **(D)**. Scale bar = 400 μm. **(E)** MTS assay illustrating the hPDLCs capture ability on different microspheres (*n* = 3). ***p < 0.001 and ****p < 0.0001.

Fluorescent dye DiI was used to pre-stain cells for improved observation, which were then co-incubated with microspheres for 6 h. The results showed that p75NTR/nHA/CS and p75NTR/CS groups captured numerous cells, whereas the nHA/CS and CS groups had a considerably lower amount (Figures 3A–D). Furthermore, Figure 3E displayed that p75NTR/nHA/CS and p75NTR/CS captured more cells than the nHA/CS and CS groups, as indicated by the MTS assay. There was no significant difference found between the p75NTR/nHA/CS group and the p75NTR/CS group (*p >* 0.05). Although the OD value in the nHA/CS group was higher than in the CS group, the difference was not statistically significant (*p >* 0.05). These results provide preliminary evidence that the p75NTR antibody can aid microspheres in capturing hPDLCs, and the presence of nHA does not noticeably affect the cell capture outcome.

### 3.4 Expression of p75NTR in cells post-capture

**FIGURE 4:**
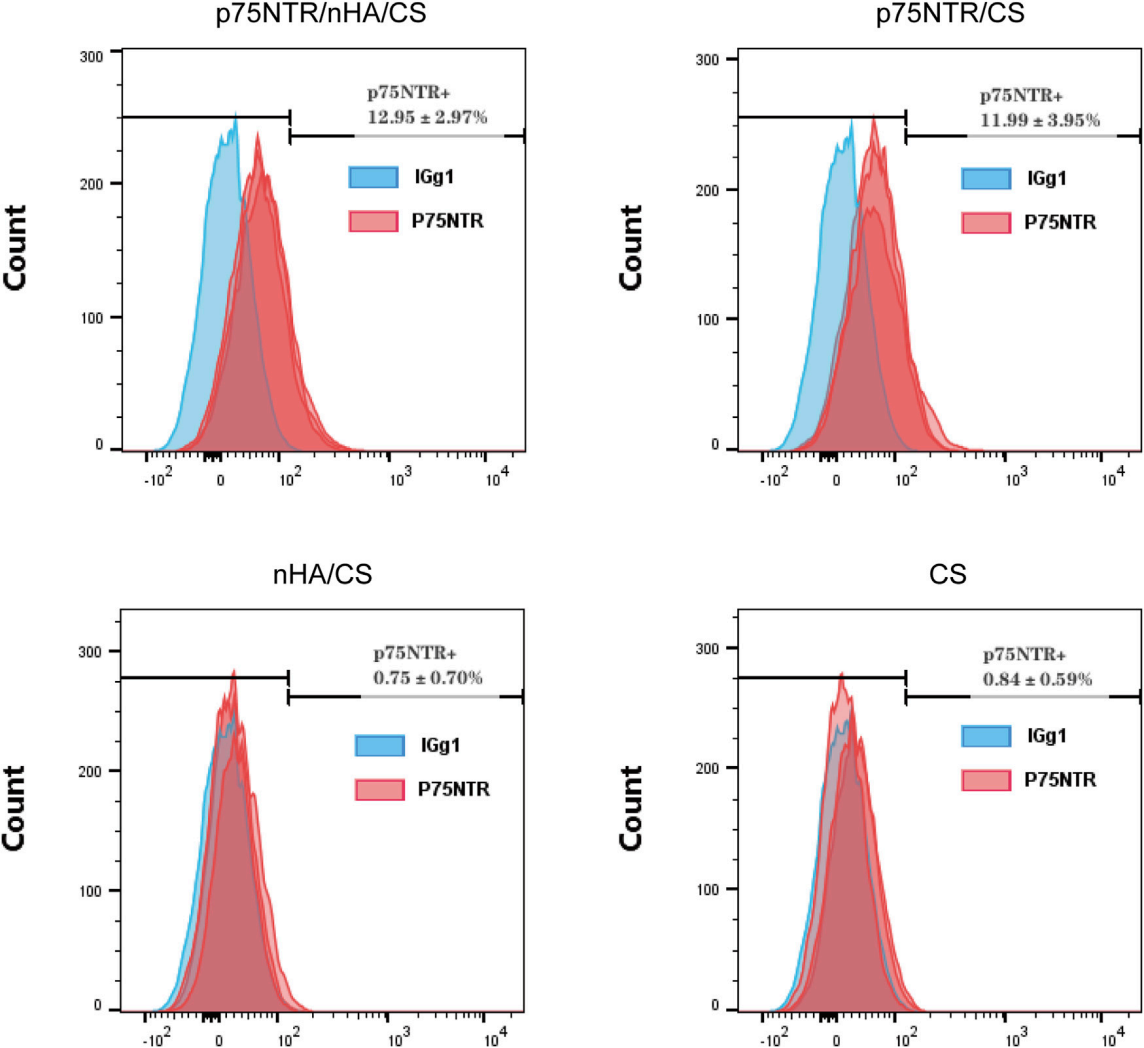
Expression of p75NTR in cells post-capture. Flow cytometry analysis of p75NTR expression in hPDLCs captured by different microspheres (*n* = 3).

Cells captured by each group of microspheres were cultured and flow cytometry was used to measure the expression of p75NTR. Analysis showed that the expression rate of p75NTR was 12.95% ± 2.97% in the p75NTR/nHA/CS group, 11.99% ± 3.95% in the p75NTR/CS group, 0.75% ± 0.70% in the nHA/CS group, and 0.84% ± 0.59% in the CS group (Figure 4). This indicates that the p75NTR expression rate in p75NTR/nHA/CS and p75NTR/CS groups significantly surpassed those in CS and nHA/CS groups. Therefore, findings suggest that p75NTR antibody-conjugated microspheres can effectively recruit p75NTR^+^hPDLCs.

### 3.5 Cell proliferation assay

**FIGURE 5:**
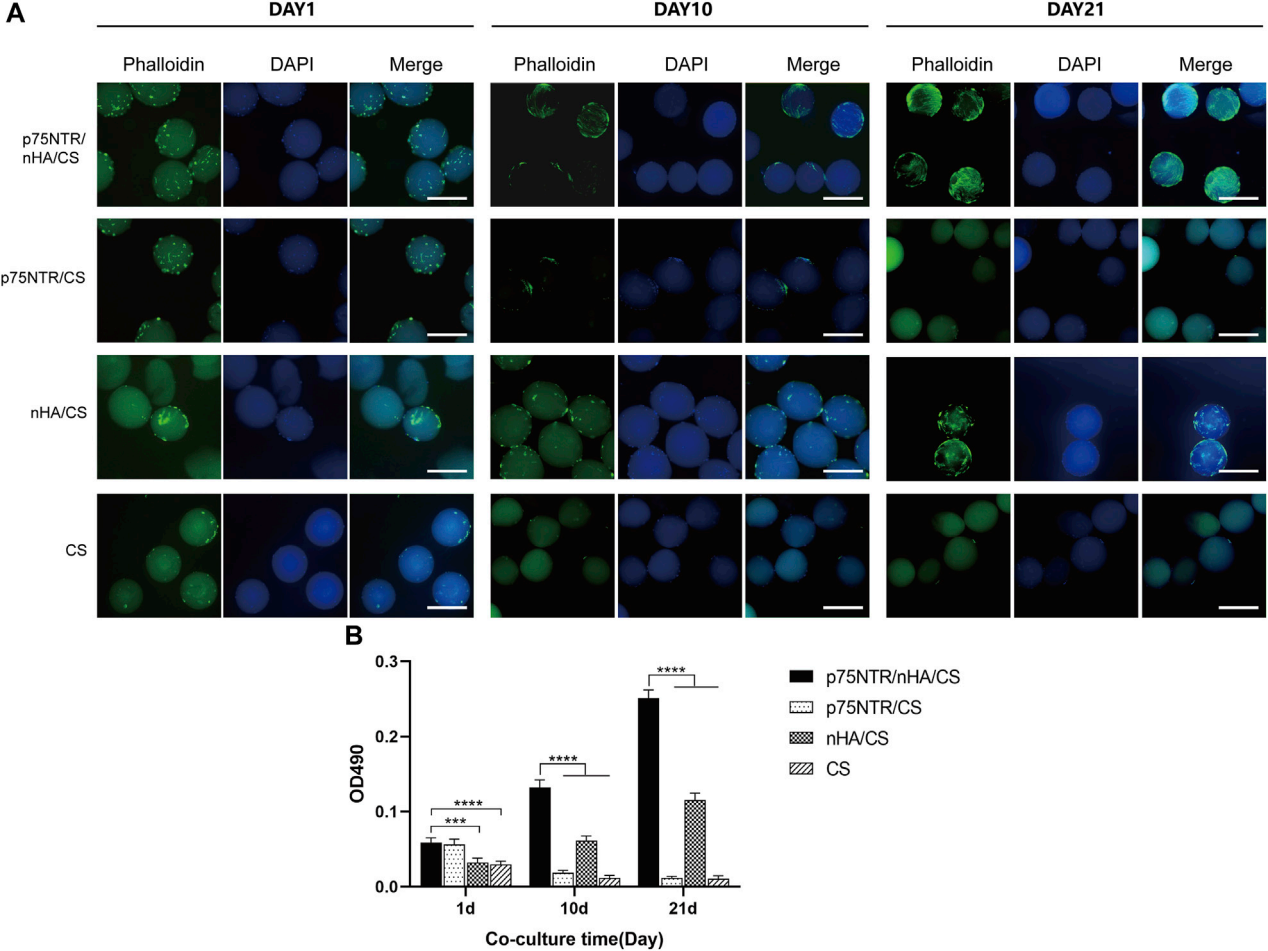
Cell proliferation on microspheres. **(A)** Fluorescent images of hPDLCs on p75NTR/nHA/CS, p75NTR/CS, nHA/CS and CS microspheres after seeding for 1 day, 10 days, and 21 days. Scale bar = 400 μm. **(B)** MTS assay of hPDLC proliferation in different microspheres (*n* = 4). Scale bar = 400 μm ***p < 0.001 and ****p < 0.0001.

The morphology of the cells on the microspheres was confirmed by DAPI and FITC-phalloidin staining at day 1, 10, and 21. Figure 5A shows that the morphology of the cells in the p75NTR/CS microspheres and the CS microspheres were globose. Furthermore, the number of cells in the p75NTR/CS and CS groups gradually decreased. At day 10, only a few cells were visible, and at day 21, the cells were barely discernible, failing to adhere effectively to the surface of the microsphere. However, in the p75NTR/nHA/CS group, cells adhered to the microspheres and proliferation in the spindle morphology was observed at day 10 and 21. Supplementary Figure S2 shows the high-magnification fluorescent images of cell proliferation. In the nHA/CS group, cell expansion occurred gradually, but the cell count at all time points was lower than that in the p75NTR/nHA/CS group. It is likely due to the fact that the initial number of cells recruited in the nHA/CS group was less than that in the p75NTR/nHA/CS group (Figure 3E). This difference in initial cell numbers could have resulted in a slower proliferation rate in the nHA/CS group compared to the p75NTR/nHA/CS group.

The MTS assay showed similar results (Figure 5B). The OD values of the p75NTR/nHA/CS and nHA/CS groups gradually increased with prolonged cultivation. The OD value at day 21 for the p75NTR/nHA/CS group was almost five times than at day 1. The cell proliferation speed in nHA/CS group was slower than p75NTR/nHA/CS group, and at each time point, the OD values of nHA/CS group were notably lower. However, the OD values of the p75NTR/CS and CS groups gradually decreased over time, with the p75NTR/CS group falling to a level comparable to the CS group at day 21. These results suggested that cells can adhere and proliferate more effectively on the surface of p75NTR/nHA/CS microspheres.

### 3.6 Osteogenic potential of microspheres

**FIGURE 6:**
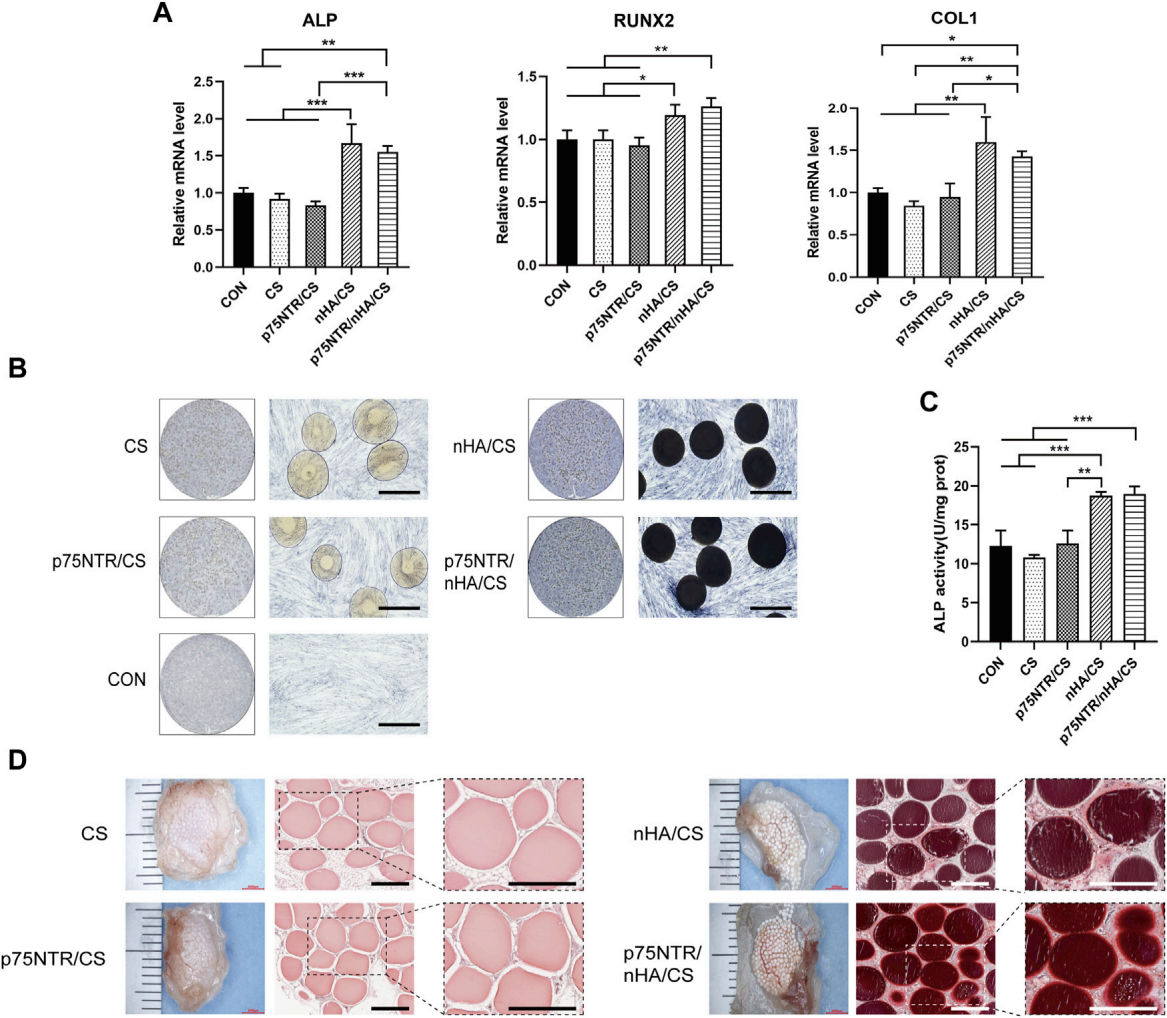
Osteogenic Potential of Microspheres. **(A–C)** hPDLCs co-cultured with microspheres were treated with osteogenic induction medium. **(A)** RT-PCR detection of *in vitro* expression of bone-specific genes *ALP*, *RUNX2*, and *COL1* at day 7 (*n* = 3; *p < 0.05, **p < 0.01 and ***p < 0.001). **(B)** ALP staining was performed at day 7. Scale bar = 400 μm. **(C)** Quantitative analysis of ALP was measured at day 7 (*n* = 3; **p < 0.01 and ***p < 0.001). **(D)** Calcium depositions around subcutaneous hPDLCs-seeded scaffolds seeded with hPDLCs were observed after alizarin red staining. Scale bar = 400 μm.

To assess the potential of microspheres to induce osteogenesis *in vitro*, microspheres and hPDLCs were co-cultured in an osteogenic induction medium. After 7 days, qRT-PCR analysis showed enhanced mRNA levels of *ALP*, *COL1*, and *RUNX2* in both the p75NTR/nHA/CS and nHA/CS groups (Figure 6A). However, there were no significant differences between the p75NTR/nHA/CS group and the nHA/CS group (*p >* 0.05). Furthermore, there was no statistically significant difference observed for the p75NTR/CS, CS, and CON groups (*p* > 0.05). Quantitative analysis of ALP activity (Figure 6C) displayed that the CON, CS, p75NTR/CS, nHA/CS and p75NTR/nHA/CS groups were 12.29 ± 1.98, 10.80 ± 0.34, 12.62 ± 1.65, 18.75 ± 0.49 and 18.94 ± 0.99, respectively. It indicated a significant increase in ALP activity in both the p75NTR/nHA/CS group and nHA/CS group compared to the other groups. Likewise, ALP staining results (Figure 6B) showed that the p75NTR/nHA/CS and nHA/CS groups exhibited more intense staining than the other groups. Moreover, the alkaline phosphatase activity is stronger around the microspheres in the p75NTR/nHA/CS group. These results suggested that the p75NTR^+^ hPDLCs recruited by the microspheres possess higher alkaline phosphatase activity and nHA can enhance the osteogenic potential of these microspheres for hPDLCs *in vitro*.

We conducted further analysis of the effectiveness of microspheres in promoting *in vivo* osteogenic differentiation of cells. After co-culturing microspheres and hPDLCs for 7 days, we implanted them in nude mice. Three weeks later, we observed the deposition of the calcified matrix using alizarin red staining (Figure 6C). Calcified nodules were present in both the p75NTR/nHA/CS and nHA/CS groups. These nodules were partially deposited on the surface of the p75NTR/nHA/CS microspheres, whereas in the nHA/CS group, they were deposited around the microspheres. In contrast, both p75NTR/CS and CS groups had no significant calcified nodules. Our findings support the notion that the presence of p75NTR antibody augments the recruitment of hPDLCs with greater osteogenic activity. In addition, we posit that the combination of CS microspheres and nHA amplifies osteogenic differentiation at the cellular level.

### 3.7 Cell recruitment capacity *in vivo*

**FIGURE 7:**
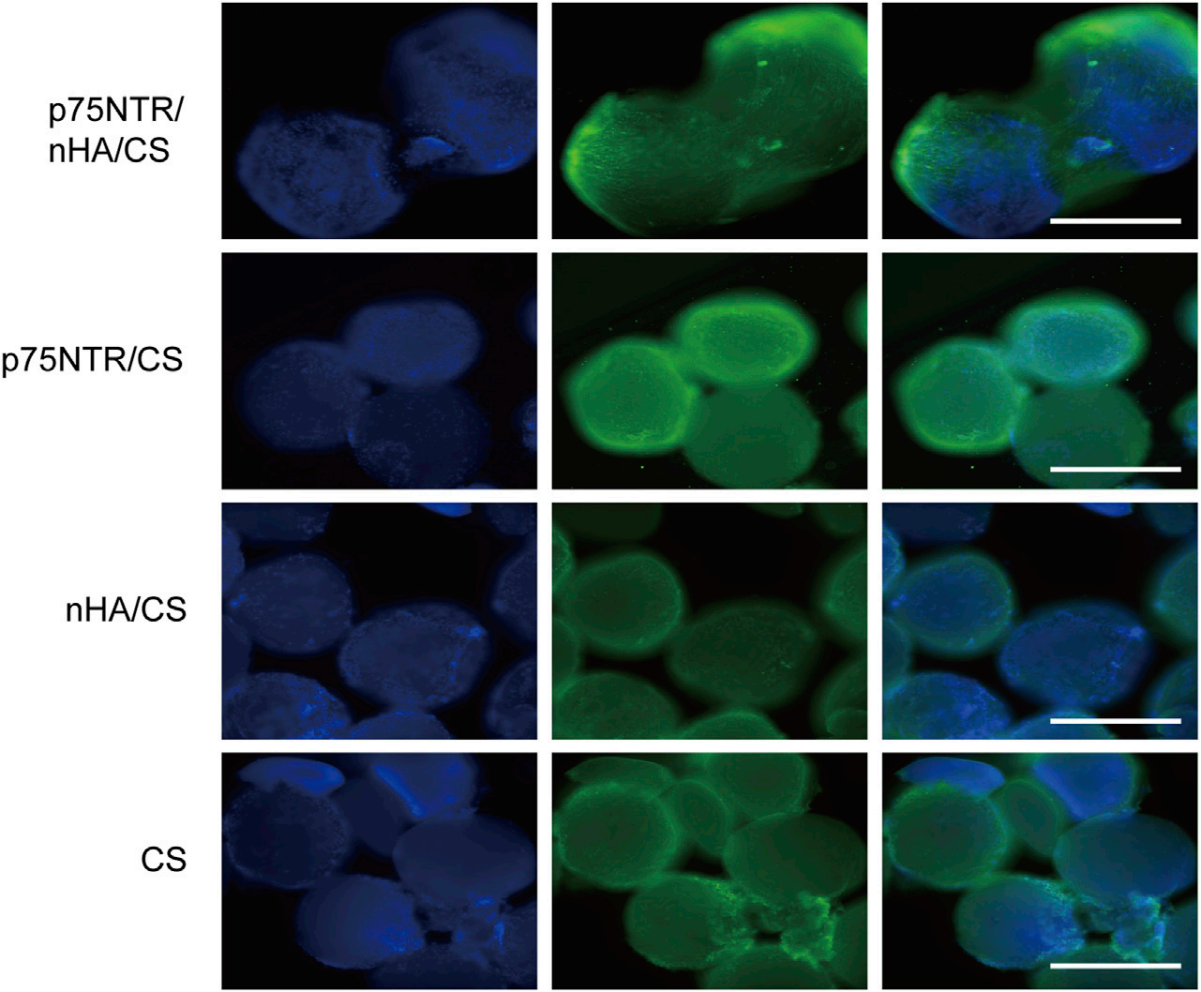
Cell recruitment *in vivo*. Immunofluorescence assays of different microspheres stained with p75NTR (green) and DAPI (blue) after implantation at day 7 *in vivo*. Scale bar = 400 μm.

Defects of alveolar bone were surgically created in rats and subsequently filled with microspheres. The aim was to evaluate the microspheres’ *in vivo* efficacy. At 7 days post-operation, we harvested the microspheres and performed immunofluorescent staining using p75NTR antibody and DAPI aiming to observe the recruitment of p75NTR^+^hPDLCs on microspheres. Green fluorescence was observed around the p75NTR/nHA/CS microspheres, indicating a substantial recruitment of p75NTR^+^ cells (Figure 7). Fewer cells were observed on the surface of the p75NTR/CS microspheres, which is consistent with *in vitro* research and probably due to the smoother surface of the CS microspheres that discourages cell adhesion and proliferation. Blue fluorescent spots indicating the presence of cells were observed on the surfaces of both nHA/CS and CS microspheres. However, the green fluorescence was not prominent, suggesting that the recruited cells were not p75NTR^+^ cells, possibly due to non-specific adhesion of the microspheres. Preliminary results support the role of p75NTR antibodies *in vivo* cell recruitment.

## 4 Discussion

In recent years, there has been a growing interest in the application of bone tissue engineering strategies in dentistry. Several scholars have conducted studies on functional composite materials to address alveolar bone defects (Wen et al., 2017). However, *in situ* tissue engineering–a method that selectively recruits endogenous cells, which avoids the disadvantages related to exogenous cell implantation-remains scarcely explored with regard to the repair of alveolar bone defects. In our study, we used the biotin-SAV pairing approach to develop nHA/CS microspheres conjugated with p75NTR antibodies. These microspheres can attract hPDLCs with potent regenerative properties and stimulate osteogenesis.

The biotin-SAV interaction is widely acknowledged as one of the most robust noncovalent bonds in nature, with an estimated dissociation constant (Kd) of approximately 10^−14^ M (González et al., 1997). It is commonly used to bond a variety of biomolecules chemically to material surfaces. In this study, the primary amine groups (-NH_2_) existing on the chitosan (CS) microsphere surface can react with NHS-biotin in a weakly basic buffer solution (pH 7-9). This reaction creates stable amide bonds, allowing the introduction of biotin on the microsphere surface (Jiang et al., 2004). Subsequently, the biotin-SAV interaction can conjugate biotin-modified p75NTR antibodies to the microsphere surface. Antibodies can be coupled on the surfaces of both the CS and nHA/CS microspheres. Notably, owing to the greater transparency of the p75NTR/CS microspheres compared with p75NTR/nHA/CS microspheres, the immunofluorescent staining of the p75NTR/CS group is significantly more pronounced than that of the p75NTR/nHA/CS group (Figure 1).

Antibodies, with their capacity to identify and bind specific target antigens and their extended half-life *in vivo* (Dai et al., 2021; Vantourout et al., 2021), emerge as a promising method for selective recruitment of cells (Rosato et al., 2022). For example, T-cell engaging bispecific antibodies (BsAb) have been used by Niels *et al.* to concurrently bind antigens on tumor cells and the CD3 subunit on T cells, thereby selectively recruiting T cells to eliminate tumor cells (van de Donk and Zweegman, 2023). Periodontal ligament stem cells (PDLSCs) are a type of adult stem cells and originate from cranial neural crest (CNC) cells. p75NTR, a specific marker of CNC-derived stem cells, can be used to screen and purify PDLSCs. In 2015, Alvarez *et al.* isolated hPDLSCs from hPDLCs using p75NTR antibodies (Alvarez et al., 2015). Our study found that only 12.95% ± 2.97% of the cells captured by p75NTR/nHA/CS microspheres expressed p75NTR (Figure 4). The low purity of the captured cells expressing p75NTR could be explained by the microspheres’ non-specific adhesion and the loss of stemness in cell culture. Yasui et al. (2016) confirmed that stem cells inevitably undergo changes in their surface markers and biological properties throughout the *in vitro* culture, digestion and passaging processes. In addition, p75NTR has been found to play a role in osteogenic differentiation. Akiyama *et al.* demonstrated that overexpressing p75NTR in the human MG63 osteoblastic cell lines increased osteogenic differentiation (Akiyama et al., 2014). In a previous study, p75NTR^+^hPDLCs exhibited a higher potential for osteogenic differentiation (Li et al., 2020). The results of the alizarin red staining indicated that, in the nHA/CS group, calcified nodules were predominantly deposited around the microspheres, whereas in the p75NTR/nHA/CS group, the majority of calcified nodules were deposited on the microsphere surface (Figure 6). This may be linked to the recruitment of p75NTR^+^hPDLCs by p75NTR antibodies, which have a greater potential for osteogenic differentiation.

Nano-hydroxyapatite (nHA), serving as an excellent osteogenic inducer to improve mineralization, can be combined with other biomaterial scaffolds to form composite materials (Li et al., 2002). We found that nHA can enhance the osteogenic ability of microspheres toward hPDLCs, both *in vivo* and *in vitro* (Figure 6). This phenomenon may be attributed to the chemical environment resulting from the release of calcium (Ca) and phosphate (P) ions from nHA, which further induces osteogenic differentiation of cells (Xavier et al., 2015; Ding et al., 2019). Furthermore, we observed a gradual decline in cell count over time in both the p75NTR/CS and CS groups, potentially due to the smooth surface of CS microspheres, which is not conducive to cell adhesion. The gradual increase in cell number over time in both the p75NTR/nHA/CS group and the nHA/CS group (Figure 5) suggests that the incorporation of nHA has addressed the issue at hand. This can be attributed to the higher surface area and increased roughness provided by nHA, promoting better cell adhesion and cell-matrix interactions (Diamandis and Christopoulos, 1991; Dorozhkin, 2010; Sadat-Shojai et al., 2013). In addition, nHA displays enhanced surface wettability (Webster et al., 1999), leading to increased adsorption of vitronectin, an extracellular matrix protein that promotes adhesion of stem cells (Webster et al., 2000; Webster et al., 2001).

In this study, p75NTR/nHA/CS microspheres were designed to recruit hPDLCs specifically, highlighting their potential for promoting cellular adhesion, proliferation, and osteogenic differentiation. Nevertheless, a few limitations exist that require attention. First of all, the purity of p75NTR^+^ cells recruited by microspheres remains at relatively low levels, which could be ameliorated through surface modification strategies that minimize non-specific adhesion. Furthermore, the quantity of recruited cells is limited. Possible resolution measures call for the development of porous microspheres. Currently, one of the most promising methods to fabricate porous CS and its composite microspheres is through microfluidics technology (Yuan et al., 2022). Recent studies have shown that combining microfluidic emulsions with further freezing and *in-situ* thawing processes can produce porous, uniform, injectable, and shape-memory CS microspheres (Yang et al., 2022). These porous structures would provide extensive surface areas for cell adhesion, thereby increasing cell capacity and facilitating nutrient and metabolite transfer.

## Data Availability

The raw data supporting the conclusions of this article will be made available by the authors, without undue reservation.
