# Supplementary material for: p75NTR antibody-conjugated microspheres: an approach to guided tissue regeneration by selective recruitment of endogenous periodontal ligament cells
Source: Front Bioeng Biotechnol. 2024 Jan 31;12:1338029. doi: 10.3389/fbioe.2024.1338029 (PMC10864659; doi:10.3389/fbioe.2024.1338029)
Supplement: Supplementary file 1 [file DataSheet1.docx]

***Supplementary Material***


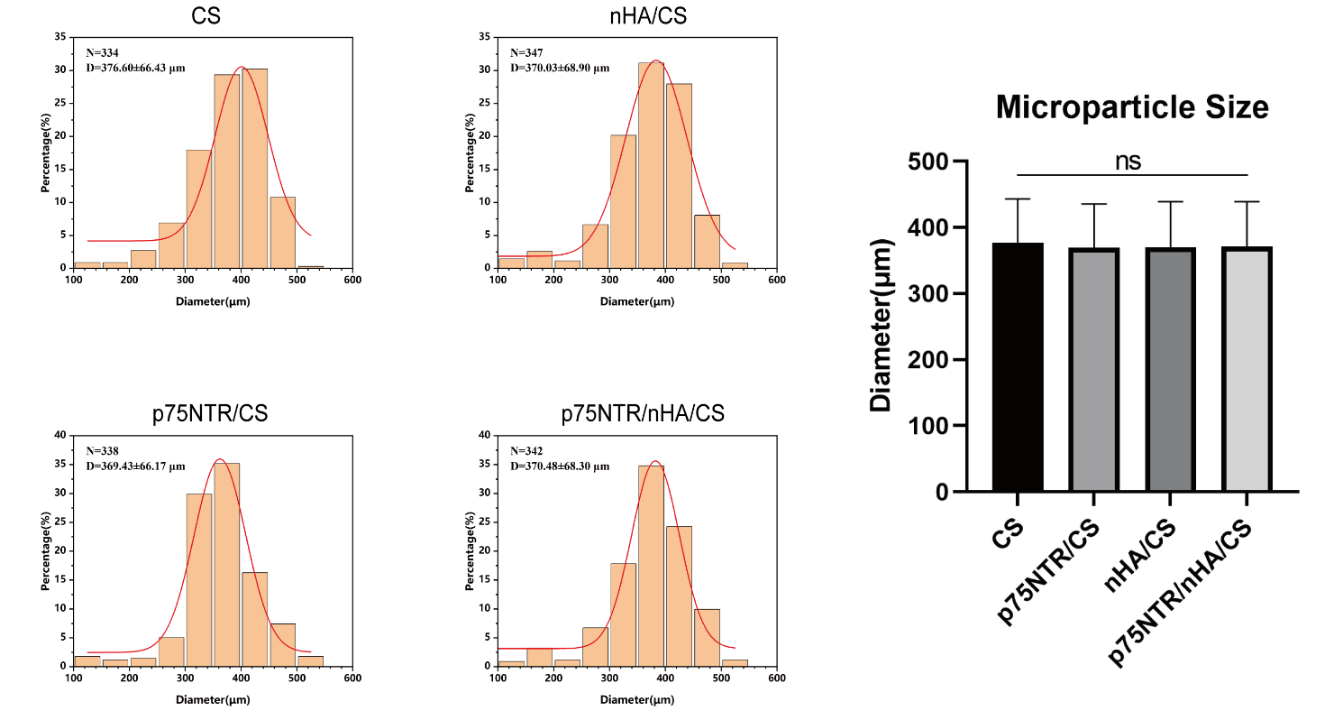


**Supplementary Figure 1.** Histogram of the distribution of microparticle size.


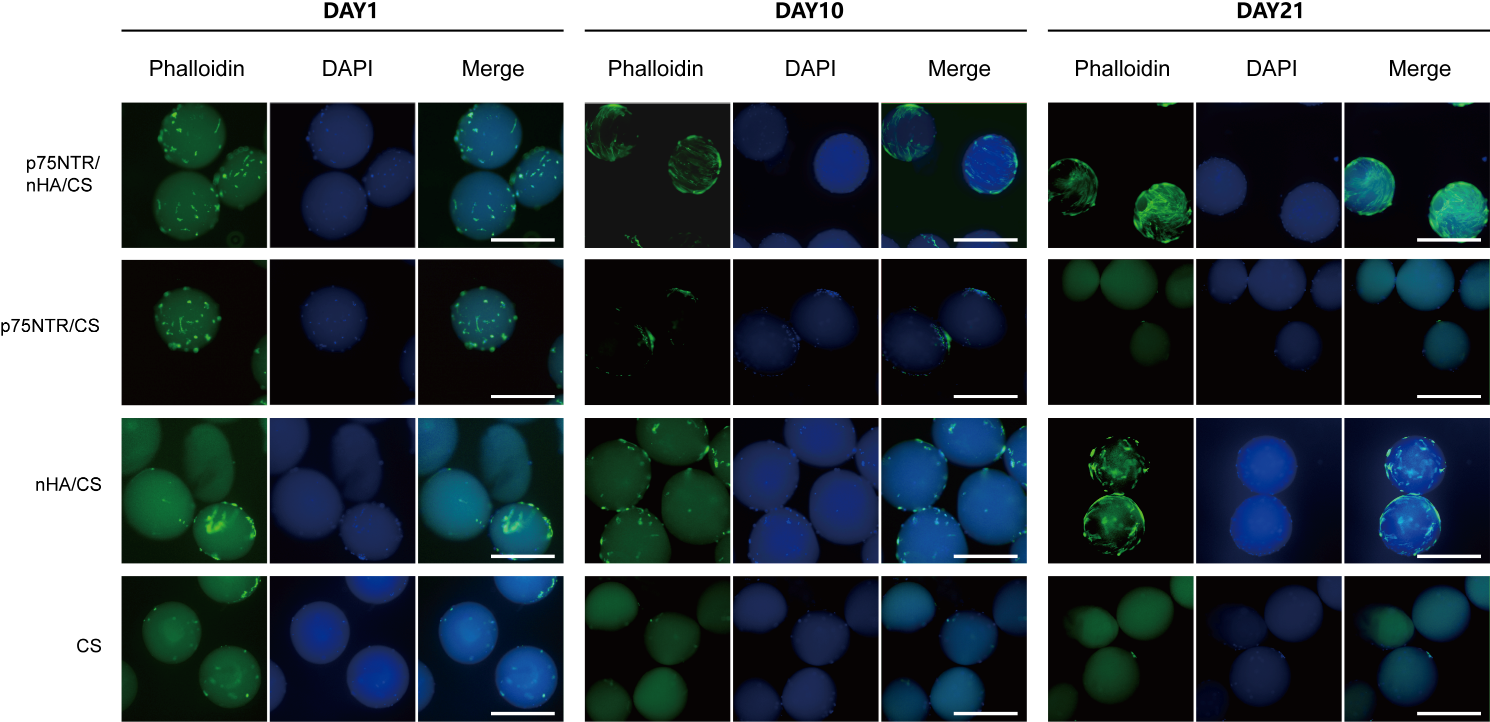


**Supplementary Figure 2.** high-magnification fluorescent images of cell proliferation stained by FITC-phalloidin and DAPI. Scale bar = 400 μm.
